# ArrayExpress update—simplifying data submissions

**DOI:** 10.1093/nar/gku1057

**Published:** 2014-10-31

**Authors:** Nikolay Kolesnikov, Emma Hastings, Maria Keays, Olga Melnichuk, Y. Amy Tang, Eleanor Williams, Miroslaw Dylag, Natalja Kurbatova, Marco Brandizi, Tony Burdett, Karyn Megy, Ekaterina Pilicheva, Gabriella Rustici, Andrew Tikhonov, Helen Parkinson, Robert Petryszak, Ugis Sarkans, Alvis Brazma

**Affiliations:** 1 European Molecular Biology Laboratory, European Bioinformatics Institute, EMBL-EBI, Wellcome Trust Genome Campus, Hinxton, CB10 1SD, UK; 2 School of Biological Sciences, Cambridge Systems Biology Centre, Tennis Court Road, Cambridge, CB2 1QR, UK

## Abstract

The ArrayExpress Archive of Functional Genomics Data (http://www.ebi.ac.uk/arrayexpress) is an international functional genomics database at the European Bioinformatics Institute (EMBL-EBI) recommended by most journals as a repository for data supporting peer-reviewed publications. It contains data from over 7000 public sequencing and 42 000 array-based studies comprising over 1.5 million assays in total. The proportion of sequencing-based submissions has grown significantly over the last few years and has doubled in the last 18 months, whilst the rate of microarray submissions is growing slightly. All data in ArrayExpress are available in the MAGE-TAB format, which allows robust linking to data analysis and visualization tools and standardized analysis. The main development over the last two years has been the release of a new data submission tool Annotare, which has reduced the average submission time almost 3-fold. In the near future, Annotare will become the only submission route into ArrayExpress, alongside MAGE-TAB format-based pipelines. ArrayExpress is a stable and highly accessed resource. Our future tasks include automation of data flows and further integration with other EMBL-EBI resources for the representation of multi-omics data.

## OVERVIEW

Established in 2003 (1), the ArrayExpress Archive of Functional Genomics Data (2) has become one of the major international repositories for microarray and high-throughput sequencing (HTS)-based functional genomics experiments. Alongside Gene Expression Omnibus (GEO) (3), it is recommended by major journals to store data supporting relevant peer-reviewed publications. Data submitted to ArrayExpress receive a permanent accession number and may remain private, i.e. accessible only to the submitter and authorized persons (such as reviewers), for a limited period of time. The data become public either when the accession number associated with the data is cited in a publication or at the user-specified release date, whichever comes first. To facilitate reproducible research (4), we promote the data compliance to the Minimum Information About a Microarray Experiment (MIAME) (5) or Minimum Information about Sequencing Experiment (MINSEQE; http://www.fged.org/projects/minseqe/) guidelines, and therefore each submission is automatically scored by these criteria allowing users to quickly identify high-quality data sets.

In addition to the data submitted directly to ArrayExpress, data from the GEO are imported in order to provide users with a single access point to functional genomics data available in the public domain. All data are available for download in a structured and standardized format, MAGE-TAB (6), which also facilitates linking to open source analysis environments such as Bioconductor (7) and GenomeSpace (http://www.genomespace.org). Moreover, where possible R objects are generated to enable users to readily manipulate the data.

For HTS data, ArrayExpress stores processed data (e.g. gene expression levels) and metadata describing the sample properties and the experimental design, and ‘brokers’ the raw sequence data to the European Nucleotide Archive (ENA) (8), linking these from ArrayExpress. For data sets that require controlled access, the raw sequence data are stored in, and should be submitted directly to, the European Genome-phenome Archive (EGA; www.ebi.ac.uk/ega).

ArrayExpress data are widely used: ∼50 GB of data are downloaded from ArrayExpress every day, by an average of 1000 different users. A recent study of a sample of around 100 peer-reviewed publications referring to ArrayExpress (9) showed that about 22% of the ArrayExpress users use our data for computational studies (e.g. meta-analyses or reproducibility), ∼20% use ArrayExpress data in combination with their own data, 28% use these data to populate value-added gene expression databases (e.g. Oncomine), whilst the remaining 30% used in bioinformatics methods development. Within the European Bioinformatics Institute, one of the main consumers of ArrayExpress data is the value-added database Expression Atlas (10), which systematically re-annotates and re-analyses data from ArrayExpress and enables gene, sample property and expression level-based queries.

Amongst the more than 50 000 studies that are in ArrayExpress, there are some key data sets that are highly accessed and downloaded for re-use or re-analysis. Examples of these core experiments are E-GEUV-1, E-MTAB-789 and E-MTAB-1733. The first is an RNA-sequencing study of 465 lymphoblastoid cell lines from the 1000 Genomes Project. ArrayExpress provides information about the study, processed data, various supplementary files and links to the raw data in ENA, as well as links to a customized RNA-sequencing data browser in Ensembl. E-MTAB-783 contains data from gene expression analysis of nearly 800 cancer cell lines using Affymetrix arrays. In the RNA-sequencing study, E-MTAB-1733 coding RNA from 27 tissues from 95 human individuals was sequenced in order to determine the tissue specificity of all protein-coding genes. This study also appears in the baseline component of the Expression Atlas at EBI (htttp://www.ebi.ac.uk/gxa/experiments/E-MTAB-1733), where one can query genes or tissue-specific expression.

The use of the Experimental Factor Ontology (EFO) (11) allows consistent query results to be returned from direct submissions as well as imported data and enables semantically driven searches, which are more powerful than keyword-driven searches.

The ArrayExpress user documentation has recently been updated and several online courses, covering how to search, interpret and submit data to ArrayExpress, can be found on the EBI e-Learning portal, Train online (http://www.ebi.ac.uk/training/online/).

## ANNOTARE SUBMISSION TOOL

A new submission tool based on the community-developed microarray data annotation tool Annotare (12) optimized for supporting microarray, as well as HTS-based data submissions, was released at the beginning of 2014. In about 6 months, over 250 submissions have been accepted to ArrayExpress via this tool. Although some development of the tool is still on-going, it has already reduced the average submission time span almost 3-fold (the submission lifetime from opening of the account to the completion of the submission for experiments with up to 100 assays has dropped from about 14 to 5 days), with a median submission time of 1 day, and 20 percent of submissions completed within 3 hours.

**Figure 1.**
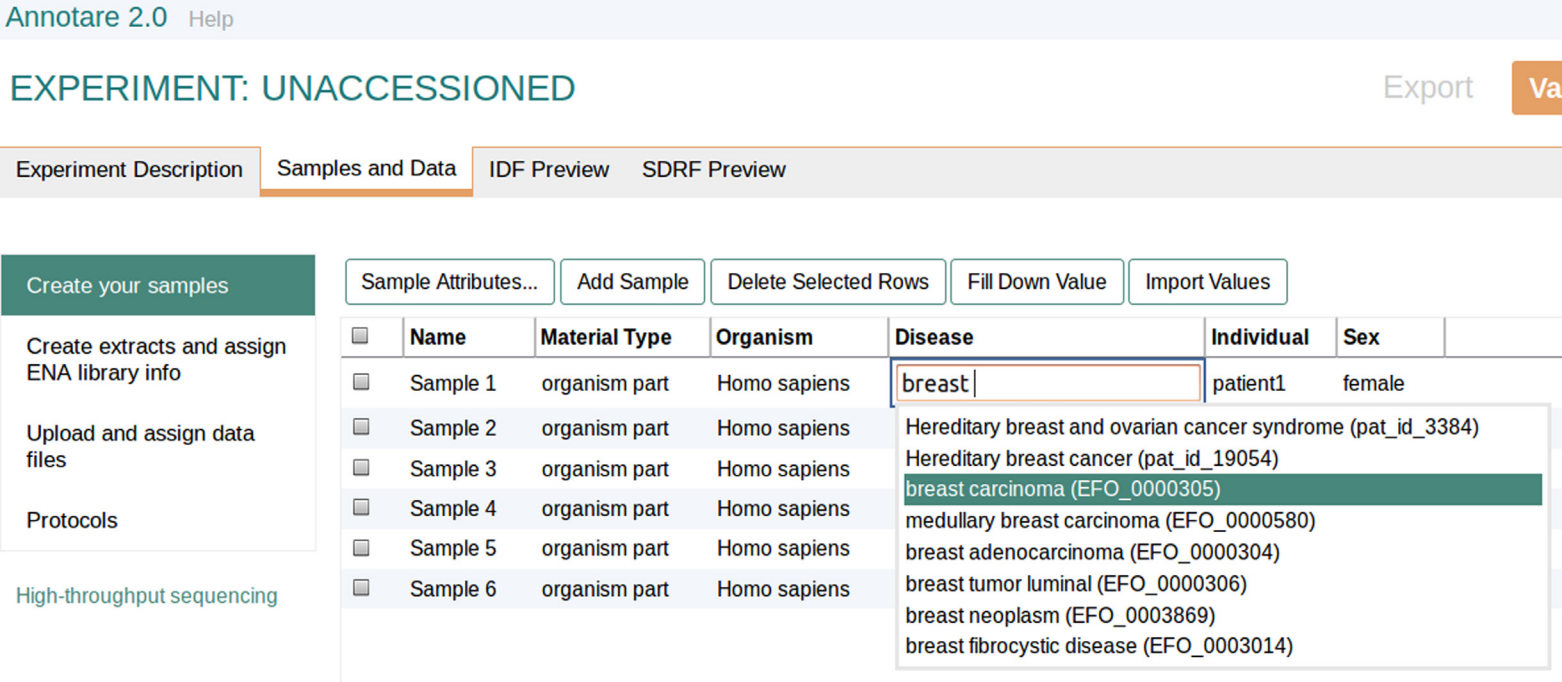
An Annotare sample submission form. In the Annotare submission system appropriate sample attributes can be chosen (e.g. disease, individual identifier, sex) and then populated either with terms selected from the Experimental Factor Ontology (EFO) or using free text. EFO terms are suggested as the user starts to type in a field. Samples can be easily added or deleted. Following sample creation submitters are guided through the assignment of labelling information for nucleic acid extracts in microarray submissions or library information such as the library layout in high-throughput sequencing submissions. Finally, users are guided through the submission of their data files.

Annotare uploads the data files from the submitter's directory and captures experimental metadata through a series of spreadsheet-based web forms (see Figure 1), guiding the submitter step by step when constructing a submission. To allow efficient population of the forms, auto fill-down and copy-and-paste functionalities have been implemented, which are particularly useful for experiments with large numbers of samples. For instance, if many samples in the experiment are of the same species, the species field needs to be filled only for the first sample, whilst the others can be propagated down with a single click. Standard terms from EFO are offered in dropdown lists where possible, to encourage the use of standardized vocabulary at the point of submission. A validation step is built in to check all the information and files provided prior to executing the submission. The validation step would catch errors such as missing data files for an assay or the absence of attributes for samples, at which point the submitter can make amendments. After validation, Annotare generates MAGE-TAB files, which contain the experiment's metadata, and submits these together with the data files to ArrayExpress, where the accession number is provided to the submitter.

Annotare has many advantages over our previous submission system. First, no prior knowledge of the MAGE-TAB format is required when preparing a submission and the risk of typographic or syntactic errors is eliminated. Second, mandatory fields are clearly indicated, allowing submitters to correct most metadata issues prior to submission and thus speeding up the process. Third, the integrated contextual help in the Annotare interface offers a smoother submission experience. But most importantly, as the decrease in the submission times and the user feedback suggest, the introduction of Annotare has significantly simplified and speeded up the submission process for the user, particularly for users without expert bioinformatics support who remain a significant proportion of the depositors.

## OTHER DEVELOPMENTS

From the user perspective, the ArrayExpress query interface has intentionally remained stable. Over the last two years (in addition to Annotare), most of the development work has been devoted to delivery of a stable data flow framework, for instance making the links and the flow of raw sequence data between ArrayExpress and the ENA seamless so users deal with a single database and set of curators, rather than multiple submission tools and processes. Minor interface improvements have been implemented, for example, from 2014 submitters can log into their data sets to change the details of the publication references associated with their submissions or to adjust the release dates. Providing greater control to users over data visibility has resulted in faster data release and freed curator time to process the increased submission numbers for RNA sequencing. The query interface has also been harmonized with all the other major EBI resources. We also now query Europe PMC (http://europepmc.org/) on a weekly basis to mine for publications that mention ArrayExpress accessions using Europe PMC's Accession Number Annotation service (13). The publication details returned are then added to the experiment record in ArrayExpress to provide an enhanced query experience.

## FUTURE DEVELOPMENTS

As HTS approaches are gradually becoming the tool of choice for functional genomics experiments, we do not envisage major ArrayExpress developments for dealing with microarray data. The main future goal of ArrayExpress will be delivery of deeper integration with the rest of the EBI resources, in particular with BioSample Database (14), which will become the authoritative source of all sample information associated with the data at EBI, with ENA, which stores the raw sequence data, European Genome-phenome Database for controlled access data, as well as the value-added database Expression Atlas, which is a major ArrayExpress data ‘consumer’ at EBI. The data flow into ArrayExpress will become increasingly automated, with the curation effort spent exclusively on data sets selected for populating the Expression Atlas.

An important role in this data integration will be played by a new resource at EBI—BioStudies database, which will serve as the hub for a wide range of different types of experiments, concentrating specifically on multi-omics experiments and unstructured data supporting publications.

## References

[1] Brazma A.P.H., Sarkans U., Shojatalab M., Vilo J., Abeygunawardena N., Holloway E., Kapushesky M., Kemmeren P., Lara G.G., Oezcimen A. (2003). ArrayExpress—a public repository for microarray gene expression data at the EBI. Nucleic Acids Res..

[2] Rustici G., Kolesnikov N., Brandizi M., Burdett T., Dylag M., Emam I., Farne A., Hastings E., Ison J., Keays M. (2013). ArrayExpress update—trends in database growth and links to data analysis tools. Nucleic Acids Res..

[3] Barrett T., Wilhite S.E., Ledoux P., Evangelista C., Kim I.F., Tomashevsky M., Marshall K.A., Phillippy K.H., Sherman P.M., Holko M. (2013). NCBI GEO: archive for functional genomics data sets—update. Nucleic Acids Res..

[4] Ioannidis J.P., Allison D.B., Ball C.A., Coulibaly I., Cui X., Culhane A.C., Falchi M., Furlanello C., Game L., Jurman G. (2009). Repeatability of published microarray gene expression analyses. Nat. Genet..

[5] Brazma A., Hingamp P., Quackenbush J., Sherlock G., Spellman P., Stoeckert C., Aach J., Ansorge W., Ball C.A., Causton H.C. (2001). Minimum information about a microarray experiment (MIAME)-toward standards for microarray data. Nat. Genet..

[6] Rayner T.F., Rocca-Serra P., Spellman P.T., Causton H.C., Farne A., Holloway E., Irizarry R.A., Liu J., Maier D.S., Miller M. (2006). A simple spreadsheet-based, MIAME-supportive format for microarray data: MAGE-TAB. BMC Bioinformatics.

[7] Gentleman R.C., Carey V.J., Bates D.M., Bolstad B., Dettling M., Dudoit S., Ellis B., Gautier L., Ge Y., Gentry J. (2004). Bioconductor: open software development for computational biology and bioinformatics. Genome Biol..

[8] Cochrane G., Akhtar R., Bonfield J., Bower L., Demiralp F., Faruque N., Gibson R., Hoad G., Hubbard T., Hunter C. (2009). Petabyte-scale innovations at the European Nucleotide Archive. Nucleic Acids Res..

[9] Rung J., Brazma A. (2013). Reuse of public genome-wide gene expression data. Nat. Rev. Genet..

[10] Petryszak R., Burdett T., Fiorelli B., Fonseca N.A., Gonzalez-Porta M., Hastings E., Huber W., Jupp S., Keays M., Kryvych N. (2014). Expression Atlas update—a database of gene and transcript expression from microarray- and sequencing-based functional genomics experiments. Nucleic Acids Res..

[11] Malone J., Holloway E., Adamusiak T., Kapushesky M., Zheng J., Kolesnikov N., Zhukova A., Brazma A., Parkinson H. (2010). Modeling sample variables with an Experimental Factor Ontology. Bioinformatics.

[12] Shankar R1 P.H., Burdett T., Hastings E., Liu J., Miller M., Srinivasa R., White J., Brazma A., Sherlock G., Stoeckert C.J. (2010). Annotare—a tool for annotating high-throughput biomedical investigations and resulting data. Bioinformatics.

[13] Kafkas S., Kim J.H., McEntyre J.R. (2013). Database citation in full text biomedical articles. PloS one.

[14] Faulconbridge A., Burdett T., Brandizi M., Gostev M., Pereira R., Vasant D., Sarkans U., Brazma A., Parkinson H. (2014). Updates to BioSamples database at European Bioinformatics Institute. Nucleic Acids Res..

